# Transfer and analysis of *Salmonella pdu* genes in a range of Gram‐negative bacteria demonstrate exogenous microcompartment expression across a variety of species

**DOI:** 10.1111/1751-7915.12863

**Published:** 2017-10-02

**Authors:** Laura Graf, Kent Wu, James W. Wilson

**Affiliations:** ^1^ Biology Department Villanova University Mendel Hall, 800 Lancaster Avenue Villanova PA 19085 USA

## Abstract

Bacterial microcompartments (MCPs) are protein organelles that typically house toxic or volatile reaction intermediates involved in metabolic pathways. Engineering bacteria to express exogenous MCPs will allow these cells to gain useful functions involving molecule compartmentalization. We cloned a 38 kb region from the *Salmonella enterica* serovar Typhimurium genome containing the *pdu* 1,2 propanediol (1,2 PD) utilization and *cob/cbi* genes using the FRT‐Capture strategy to clone and transfer large genomic segments. We transferred this clone to a range of Gram‐negative bacteria and found the clone to be functional for 1,2 PD metabolism in a variety of species including *S*. Typhimurium Δ*pdu*,* Escherichia coli, Salmonella bongori, Klebsiella pneumoniae, Cronobacter sakazakii, Serratia marcescens*, and different *Pseudomonas* species. We successfully isolated MCPs expressed from the clone from several, but not all, of these strains, and we observed this utilizing a range of different media and in the absence of protease inhibitor. We also present a mini‐prep protocol that allows rapid, small‐scale screening of strains for MCP production. To date, this is the first analysis of cloned, exogenous microcompartment expression across several different Gram‐negative backgrounds and provides a foundation for MCP use in a variety of bacterial species using a full, intact clone.

## Introduction

Bacterial microcompartments (MCPs) are protein‐based organelles that serve to compartmentalize certain metabolic reactions utilizing toxic or volatile molecules (Chowdhury *et al*., 2014; Bobik *et al*., 2015). The MCPs are generally composed of proteins that form an outer shell that is selectively permeable to house targeted enzymes and metabolic substrates and intermediates (Chowdhury *et al*., 2014, 2015; Bobik *et al*., 2015). Examples of characterized systems that utilize MCPs are carboxysomes for CO_2_ fixation in cyanobacteria and other species, 1,2 propanediol utilization in various enteric bacteria, and the ethanolamine utilization pathway expressed by a number of diverse bacteria (Chowdhury *et al*., 2014; Bobik *et al*., 2015). Several other MCP systems proposed to be involved with a range of other metabolic pathways have also been identified via genomics and bioinformatics (Abdul‐rahman *et al*., 2013; Jorda *et al*., 2013; Axen *et al*., 2014; Chowdhury *et al*., 2014; Bobik *et al*., 2015). The nature of MCPs makes them amenable for bacterial engineering in different biotechnology applications such as the ability to utilize novel metabolism, compartmentalize toxic or volatile molecules for downstream applications, express nanoscale bioreactors and potentially facilitate vaccine design strategies (Tsai and Yeates, 2011; Chen and Silver, 2012; Kim and Tullman‐Ercek, 2013; Chowdhury *et al*., 2014; Bobik *et al*., 2015).

**Figure 1:**
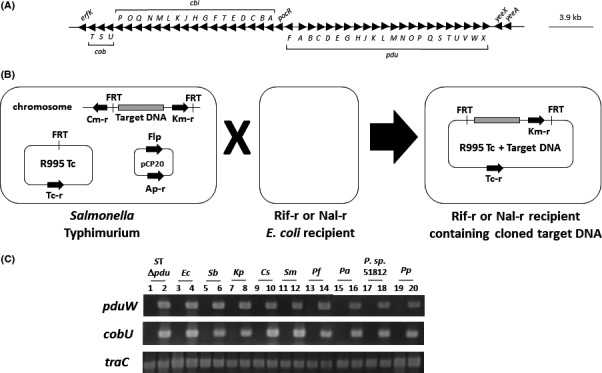
Cloning of the *S*. Typhimurium *pdu* and *cob/cbi* genes using FRT‐Capture. Panel A: A gene map of the targeted *S*. Typhimurium *pdu* and *cob/cbi* genomic segment cloned via FRT‐Capture. Panel B: A schematic diagram of the FRT‐Capture technique (not drawn to scale). FRT sites were inserted into regions flanking the target DNA (i.e. the *pdu*/*cob/cbi* region) in *S*. Typhimurium. Plasmids R995 (containing a single FRT site) and pCP20 (expressing FLP recombinase) were sequentially introduced into this strain. This allows FLP‐mediated excision of the target DNA and insertion into R995. The cloned target DNA construct is isolated upon conjugation to a fresh *E. coli* recipient strain containing appropriate counterselection (such as Rif‐R or Nal‐R). Panel C: PCR analysis of R995 + *pdu* *S*T in a range of Gram‐negative bacterial backgrounds. The *pduW* and *cobU* genes are specific for the cloned DNA segment, and the *traC* gene is present on the R995 vector. PCR products were analysed using 1.5% agarose gel electrophoresis followed by staining with SYBR Safe and UV light visualization. Abbreviations: *S*T Δ*pdu* = *Salmonella* Typhimurium Δ*pdu*;* Ec* = *Escherichia coli*;* Sb* = *Salmonella bongori; Kp* = *Klebsiella pneumoniae*;* Cs* = *Cronobacter sakazakii*;* Sm* = *Serratia marcescens*;* Pf* = *Pseudomonas fluorescens; Pa = Pseudomonas aeruginosa; P. sp*. 51812 = *Pseudomonas* species 51812; *Pp* = *Pseudomonas putida*. Odd numbered lanes are R995‐containing, and even numbered lanes are R995 + *pdu* *S*T‐containing.

The shell of the *S*. Typhimurium Pdu MCP is composed of protein subunits PduA, B, J, K, M, N, T and U (Bobik *et al*., 1999, 2015; Cheng *et al*., 2011; Chowdhury *et al*., 2014; Sinha *et al*., 2014; Jorda *et al*., 2015). PduA appears to play a central role in forming a selectively permeable pore that allows 1,2 PD entry into the MCP while restricting escape of the toxic compound propionaldehyde (Chowdhury *et al*., 2015). The enzymes for the 1, 2 PD utilization pathway are PduC, D, E, L, P, Q and W (Chowdhury *et al*., 2014; Bobik *et al*., 2015). The metabolism of 1, 2 PD requires coenzyme B_12_, and therefore, enzymes involved with coenzyme B_12_ assimilation and recycling are also associated with the Pdu MCP (Johnson *et al*., 2001; Sampson *et al*., 2005; Fan *et al*., 2009; Chowdhury *et al*., 2014; Bobik *et al*., 2015). The structural and regulatory genes for the Pdu MCP are found in the *S*. Typhimurium chromosome on a contiguous 23‐gene segment that is immediately adjacent to the *cob/cbi* genes used for synthesis of coenzyme B_12_ (on a contiguous 20‐gene segment) (Fig. 1A) (Bobik *et al*., 1999; McClelland *et al*., 2001).

Cloning and transfer of MCP‐encoding genes would allow engineering of target bacterial strains (potentially in a range of genera) to achieve novel MCP‐related functions as discussed above. In addition, such experiments allow an evolutionary analysis of *pdu* genes in which we can ask: Are the *pdu* genes from a given species expressed and functional in any other genera/species as opposed to having evolved to be restricted in these functions to the species of origin? Previous reports describing cloned Pdu MCP genes are present in the literature (Parsons *et al*., 2008; Sargent *et al*., 2013; Matsubara *et al*., 2016). Sargent et al. synthesized and cloned a subset of the *S*. Typhimurium *pdu* genes and observed MCP formation in an *Escherichia coli* background (Sargent *et al*., 2013). Parsons et al. cloned the *pdu* genes from *Citrobacter freudii* using a cosmid library approach, transferred the clone to an *E. coli* background and observed MCP formation in this species (Parsons *et al*., 2008). Matsubara et. al. cloned a subset of the *Klebsiella pneumoniae pdu* genes and coupled them with a *Shimwellia blatte* 1,2 PD synthetic pathway in *E. coli* to achieve 1‐propanol production in this background (Matsubara *et al*., 2016). In this study, we describe the first cloning of the entire, contiguous *S*. Typhimurium *pdu*/*cob/cb*i gene cluster (performed using a convenient *in vivo* approach termed FRT‐Capture) and the transfer of this clone to a range of different Gram‐negative genera to allow the first systematic analysis of MCP formation in a variety of bacterial species. An advantage of the present approach is the use of the entire contiguous genomic segment that contains the endogenous transcriptional and translational sequences with associated regulatory sequences. We report MCP expression from this clone across a range of bacterial genera and what appears to be robust recovery of MCPs from sample permissive strains using a range of different bacterial media.

## Results

### Cloning of the intact *S*. Typhimurium *pdu/cob/cbi* gene segment

To study Pdu MCP formation across bacterial genera for potential bacterial engineering purposes, we cloned a contiguous 38 kb segment of the *S*. Typhimurium genome containing the *pdu* and *cob/cbi* genes (please see Fig. 1 and Experimental Procedures for details). We used the FRT‐Capture method which allows *in vivo* cloning of large DNA segments and utilizes the broad host range plasmid vector R995 for convenient transfer of the clone to a wide variety of Gram‐negative bacteria (Santiago *et al*., 2011; Wilson *et al*., 2013). We transferred the R995 + *pdu S*T clone (or R995 as vector control) to a range of Gram‐negative strains including *S*. Typhimurium Δ*pdu*,* Escherichia coli, Salmonella bongori, Klebsiella pneumoniae, Cronobacter sakazakii, Serratia marcescens,* and 4 species of *Pseudomonas* (*P. aeruginosa, P. fluorescens, P. putida,* and *P. species* 51812). Plasmid DNA was isolated from each of the transconjugant strains, and PCR analysis was used to confirm the transfer of the correct corresponding plasmid (Fig. 1, Panel C).

### Functional expression of the *pdu* genes from R995 + *pdu S*T

**Figure 2:**
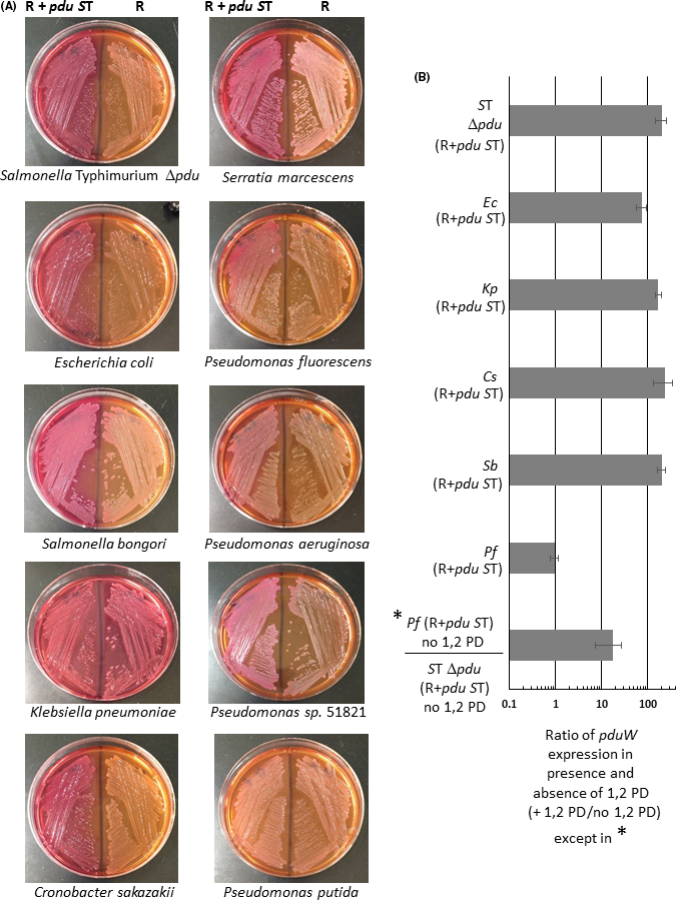
Expression analysis of *pdu* genes from R995 + *pdu* *S*T. Panel A: The indicated bacterial species containing either R995 + *pdu* *S*T or the R995 vector control were streaked onto MacConkey medium supplemented with 1,2 PD and coenzyme B_12_. Utilization of 1,2 PD will result in a pink/red colony colour on this medium. Panel B: RT‐qPCR analysis of *pdu* gene expression from R995 + *pdu* *S*T is shown. The indicated bacterial species containing R995 + *pdu* *S*T were grown in the presence or absence of 1,2 PD, and the total RNA was harvested for each sample. The RNA was converted to cDNA and then used as template in qPCR analysis with primers targeting the *pduW* or *korB* genes (the latter being a gene present on the R995 vector backbone). The qPCR product levels were normalized to the level of the *korB* gene, and a ratio of *pduW* levels in the presence and absence of 1,2 PD was calculated to give a fold difference in expression between the two growth conditions. Species abbreviations correspond to those in the Fig. 1 legend.

To demonstrate functional expression of the *pdu* genes from R995 + *pdu S*T, we plated the indicated strains on MacConkey medium containing 1,2 PD as the carbon source and supplemented with coenzyme B_12_. If 1,2 PD utilization is accomplished by any R995 + *pdu S*T strain, the result will be a visual pink/red colony colour on this medium (as compared to the corresponding control strain). For all R995 + *pdu S*T strains except *P. putida*, clearly visible pink/red colony colour was observed compared with the R995 vector control strain (Fig. 2, Panel A). For *P. putida*, the R995 + *pdu S*T and R995 strains looked virtually identical on this medium (Fig. 2, Panel A). When comparing the *K. pneumoniae* strains, we observed that the R995 control strain displayed a pink colour on this medium that was, however, less intense than that observed for the R995 + *pdu S*T strain (which was darker pink) (Fig. 2, Panel A). This is likely due to the previously documented presence of *pdu* utilization genes in the *K. pneumoniae* genome that is causing a background level of 1,2 PD metabolism (Bobik *et al*., 1997, 1999; Honjo *et al*., 2015). To test whether the 1,2 PD metabolism displayed by R995 + *pdu S*T is dependent on coenzyme B_12_, we streaked the same strains to MacConkey medium containing 1,2 PD but without coenzyme B_12_. We observed no pink colony colour for any of the strains on this medium thus confirming that the 1,2 PD utilization directed by R995 + *pdu S*T is dependent on coenzyme B_12_ as would be expected (Fig. S1). These results indicate that R995 + *pdu S*T expresses functional MCPs and endows a range of Gram‐negative bacteria the ability to metabolize 1,2 PD.

Previous studies have shown that expression of the *pdu* genes requires the presence of 1,2 PD (Bobik *et al*., 1992; Chen *et al*., 1995; Kim *et al*., 2014). To determine whether the *pdu* genes on R995 + *pdu S*T display this same pattern of expression, we performed RT‐qPCR with total RNA isolated from R995 + *pdu S*T strains grown with and without 1,2 PD. In five of the six strains tested, we observed the predicted induction of *pdu* gene transcription in the presence of 1,2 PD (Fig. 2, Panel B). In the *P. fluorescens* background, we did not observed a difference in *pdu* gene expression between the two conditions (Fig. 2, Panel B). However, when normalized signal was compared between *P. fluorescens* and *S*. Typhimurium Δ*pdu* strains (both containing R995 + *pdu S*T) under conditions without 1,2 PD, we observed 10‐fold greater signal in the *P. fluorescens* strain (Fig. 2, Panel B). This result was also obtained if the other backgrounds in the ‘without 1,2 PD’ conditions were used to compare to the *P. fluorescens* background (data not shown). This indicates that the *pdu* genes are expressed above uninduced levels in the *P. fluorescens* background in both the presence and absence of 1,2 PD. This observation suggests that the regulatory circuit that results in lower *pdu* gene expression in the absence of 1,2 PD is not working properly in the *P. fluorescens* background. It also indicates that maximal expression of the *pdu* genes in *P. fluorescens* may be about 10‐fold lower than that observed in strains where the 1,2 PD induction was observed (comparing the approximately 100‐fold induction values of the other strains to the approximately 10‐fold *P. fluorescens* value above *S*. Typhimurium Δ*pdu* in the absence of 1,2 PD). Thus, of the six strains tested, five demonstrated *pdu* gene regulation via the presence and absence of 1,2 PD while one demonstrated what appears to be (relatively lower) constitutive expression that is not altered by the presence of 1,2 PD.

### Isolation of MCPs from strains containing R995 + *pdu S*T

**Figure 3:**
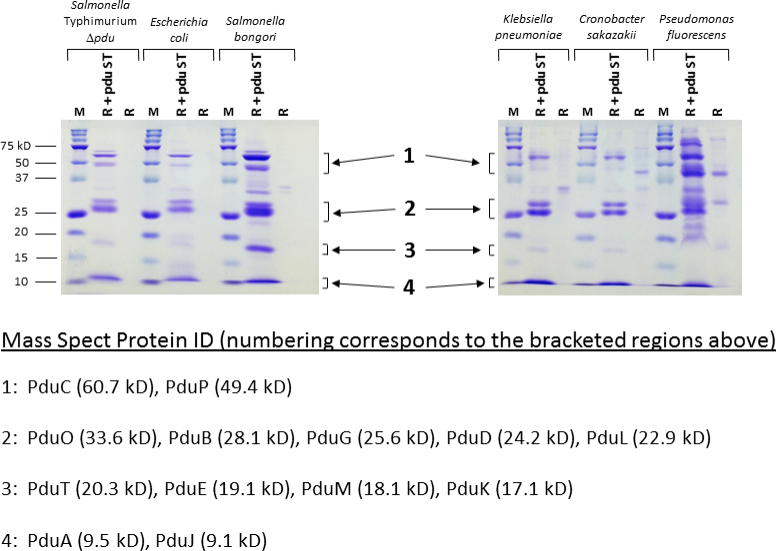
SDS‐PAGE analysis of isolated MCPs from R995 + *pdu* *S*T strains. The indicated strains containing either R995 + *pdu* *S*T or the R995 vector were grown in Pdu medium, and the MCPs were isolated from the cultures as described in Experimental Procedures. An aliquot of each MCP prep (corresponding to approximately 7–15 μg protein for the R995 + *pdu* *S*T preps) was run via SDS‐PAGE and stained with Coomassie Blue. ‘M’ stands for the protein marker (BioRad Precision Plus Kaleidoscope, 15 μg loaded). Protein band locations indicated by the numbered brackets were excised from R995 + *pdu* *S*T samples and subjected to Nano‐LC‐MS/MS mass spectrometry analysis. The protein IDs obtained from mass spectrometry analysis of samples cut from gels at the approximate indicated locations are provided as noted.

To demonstrate that the MCPs expressed from R995 + *pdu S*T could be isolated from different bacteria, we performed MCP purification using the various bacterial species containing R995 + *pdu S*T. We used a modified strategy based on a previously established MCP purification protocol (Sinha *et al*., 2012) (please see Experimental Procedures for details). This protocol uses 5.7‐fold less culture volume (70 ml versus 400 ml with the 70 ml providing approximately 1 × 10^11^ cells) and takes approximately 2 h compared with 4 h of the previous protocol with comparable yields (approximately 1 mg in both cases) (Sinha *et al*., 2012). When analysed via SDS‐PAGE and Coomassie staining, we found that the R995 + *pdu S*T MCP samples contained several bands running at sizes consistent with known *S*. Typhimurium Pdu proteins that were absent from samples obtained from corresponding R995 control strains (Fig. 3). To confirm the identity of the protein bands in the R995 + *pdu S*T samples, we subjected them to mass spectrometry analysis which positively identified *S*. Typhimurium *Pdu* proteins running at the predicted sizes (Fig. 3). The SDS‐PAGE analysis revealed purified MCPs expressed from R995 + *pdu S*T in *S*. Typhimurium Δ*pdu*,* E. coli*,* S. bongori*,* K. pneumoniae*,* C. sakazakii* and *P. fluorescens*. Intriguingly, we did not recover MCPs in preps from *S. marcescens*,* P. aeruginosa* and *P. putida* where preps displayed bands that ran at locations not corresponding to known Pdu proteins and were commonly found in the corresponding control background strains (data not shown). In the *P*. species 51812 background, we recovered MCP structures (visualized via sds‐page and electron microscopy) from both the R + *pdu S*T strain and control strain indicating that this species expresses an endogenous MCP‐like structure (which made interpretation of our MCP preps difficult for this strain background) (data not shown).

**Figure 4:**
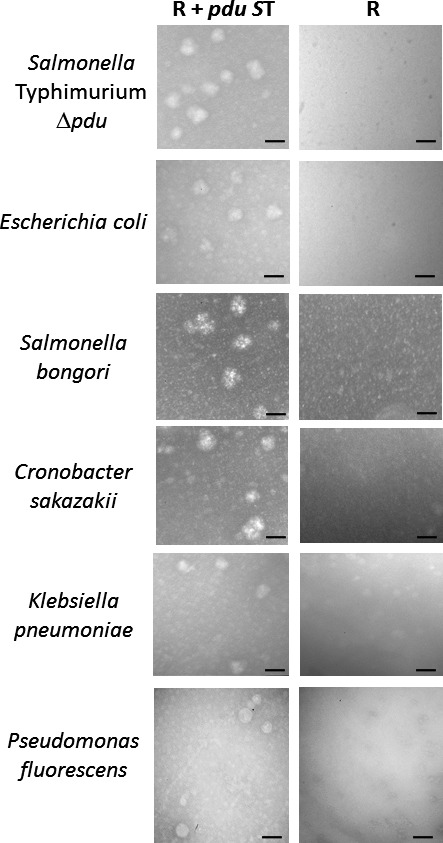
Transmission electron microscopy analysis of isolated MCPs. Samples from the indicated MCP preps were stained with 4% uranyl acetate and visualized using TEM. The size bar in each micrograph corresponds to 100 nm.

To visualize the isolated MCPs, we performed transmission electron microscopy analysis on the MCP samples (please see Experimental Procedures for details). We clearly observed MCPs in samples from *S*. Typhimurium Δ*pdu*,* E. coli*,* S. bongori*,* K. pneumoniae, C. sakazakii* and *P. fluorescens* containing R995 + *pdu S*T (Fig. 4). We did not observe these structures in samples from the same species containing the R995 vector control (Fig. 4). We measured the diameter of the purified MCP structures in the TEM images for each species, and we found the average for these measurements to be between 88–95 nm (Fig. S2). These data are consistent with previously published numbers from similar MCP measurements (Parsons *et al*., 2008; Cheng *et al*., 2011; Sinha *et al*., 2012).

### Use of different growth media reveals robust R995 + *pdu S*T MCP expression and isolation

**Figure 5:**
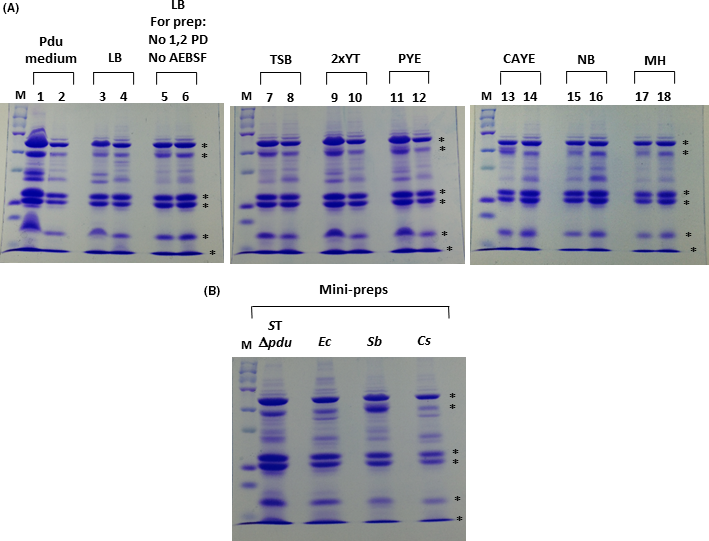
Analysis of R995 + *pdu* *S*T MCP isolation using different growth media and a mini‐prep protocol. Panel A: *S. bongori* and *C. sakazakii* strains containing R995 + *pdu* *S*T were grown in the indicated media supplemented with 1,2 PD, and the MCPs were isolated from each culture. An aliquot of each MCP prep (corresponding to approximately 10–20 μg protein) was run via SDS‐PAGE and stained with Coomassie Blue. The ‘M’ lane is protein marker (15 μg), odd numbered lanes contain samples from *S. bongori* R995 + *pdu* *S*T, and even numbered lanes contain samples from *C. sakazakii* R995 + *pdu* *S*T. Abbreviations correspond to those indicated in the Experimental Procedures for each medium. Control strains containing the R995 vector did not display MCP bands when grown and analysed in these media (data not shown). The asterisks on the right side of each gel indicate bands corresponding to known *S*. Typhimurium Pdu proteins. The sample ‘LB, For prep: no 1,2 PD, no AEBSF’ indicates MCP preps obtained from cells grown in LB medium (containing 1,2 PD) but with no 1,2 PD or protease inhibitor present during the purification procedure. Panel B: The indicated strains containing R995 + *pdu* *S*T were grown in LB medium supplemented with 1,2 PD, and the MCPs from each culture were isolated using a mini‐prep protocol as described in Experimental Procedures. An aliquot of each MCP mini‐prep was run via SDS‐PAGE and stained with Coomassie Blue. Species abbreviations correspond to those indicated in the legend for Fig. 1.

Previous reports of Pdu MCP expression and isolation primarily utilize a minimal medium for bacterial growth, presumably to assure 1,2 PD‐specific expression of the *pdu* genes in the absence of glucose (since glucose has been shown to repress *pdu* gene expression) (Ailion *et al*., 1993; Chen *et al*., 1995; Kim *et al*., 2014). This could also be due to various researchers (understandably) following established convention in the field in which minimal media were previously shown to be effective and reproducible for Pdu MCP expression. In the experimental data described in this report above, we also used a minimal medium for our initial analysis across species (termed ‘Pdu medium’). To explore the possibility that MCP expression from R995 + *pdu S*T could be obtained using rich media, we grew strains containing R995 + *pdu S*T in various rich media (supplemented with 1,2 PD) and performed MCP isolation and SDS‐PAGE analysis. The results from species *S. bongori* and *C. sakazakii* are displayed in Fig. 5, and these data indicate a robust expression and recovery of MCPs in a range of different complex media (Fig. 5). We observed similar results in these media for the other R995 + *pdu S*T strains analysed in Fig. 3 as well (see Table S2 for data from LB media). This demonstrates that the glucose levels present in these media are below the level needed to repress expression of the *pdu* genes from R995 + *pdu S*T and that there appears to be a wide flexibility for functional growth media that can be used with this clone. Previously established protocols have included both 1,2 PD and protease inhibitor to be present during the entirety of the MCP preparation (Parsons *et al*., 2008; Sinha *et al*., 2012). While the purpose of protease inhibitor is self‐explanatory, we assume that the presence of 1,2 PD during cell lysis and MCP purification is meant to stabilize the MCP in the case that any of the Pdu proteins need the initial substrate to maintain proper conformation for an intact MCP. However, we hypothesized that the MCP protein parts would be tightly interlocked such that the MCP structure (and its contents) would be inherently stable during purification such that 1,2 PD and protease inhibitor would not be necessary for the isolation protocol. Therefore, we performed the MCP isolation protocol described in Experimental Procedures in the absence of 1,2 PD and protease inhibitor for cell lysis and purification. We observed essentially identical results comparing preps performed in the presence and absence of 1,2 PD and protease inhibitor during purification further demonstrating the robust nature of MCP isolation from R995 + *pdu S*T strains (Fig. 5).

### Mini‐prep isolation of MCPs from R995 + *pdu S*T strains

We reasoned that MCP isolation from R995 + *pdu S*T strains would be amenable to a smaller scale protocol using fewer cells, smaller buffer volumes and a microfuge (so as to effectively be an MCP ‘mini‐prep’). We designed such a protocol (described in Experimental Procedures) using approximately sevenfold less cells for starting material and processed using 1.5 ml microfuge tubes in a microfuge. The mini‐prep protocol yielded comparable MCPs as compared to previous preps (though total MCP yield was lower as would be expected and discussed in Experimental Procedures) (Fig. 5). The MCP mini‐prep allows convenient MCP isolation from several cultures processed at the same time, and the mini‐prep will be applicable to other MCP systems as well (such as carboxysomes and the ethanolamine MCP).

## Discussion

We have cloned the entire, contiguous *pdu*/*cob/cbi* gene segment from the *S*. Typhimurium genome onto the plasmid vector R995 which can conveniently transfer to a range of Gram‐negative species. This allows relatively straightforward use of the Pdu MCP for cellular engineering in different bacterial backgrounds. It also allows the analysis of MCP expression, function, and isolation across species as an evolutionary study (i.e. has the *S*. Typhimurium *pdu* gene system evolved to be restricted to function in only *S*. Typhimurium or can it be functionally expressed and isolated from a range of species?). We observed functional MCP expression and isolation from *S*. Typhimurium Δ*pdu*,* E. coli, S. bongori, K. pneumoniae, C. sakazakii* and *P. fluorescens*. Interestingly, we observed MCP function in *S. marcescens* and *P. aeruginosa*, but we did not observed successful MCP isolation from these species. For some reason, it could be that the MCP is not stable enough for isolation or requires additional specific protocol treatment in these bacteria. We did not observe evidence for Pdu MCP functional expression in *P. putida*. Further study is required to determine the reason for this such as testing the transcription and translation of specific *pdu* genes on R995 + *pdu S*T in *P. putida*. In the *P*. species 51812 background, we observed the presence of copious endogenous MCP‐like structures expressed in the control strain. We do not know the nature of these structures, and further characterization is required to determine their composition and function. We found that *pdu* gene transcription from R995 + *pdu S*T in *P. fluorescens* was not regulated by the presence/absence of 1,2 PD (i.e. it appeared to be constitutively expressed regardless of 1,2 PD status). The PocR transcriptional activator (encoded in the cloned *pdu*/*cob/cbi* segment) plays a central role in the induction of *pdu* genes in the presence of 1,2 PD (Bobik *et al*., 1992; Ailion *et al*., 1993; Chen *et al*., 1995). In addition, the Crp protein is required for full induction of the *pdu* genes under both aerobic and anaerobic conditions (Bobik *et al*., 1992; Chen *et al*., 1995). It is possible that PocR and/or Crp have altered activity at the *pdu* promoter(s) in the *P. fluorescens* background such that the result is constitutive *pdu* gene expression from R995 + *pdu S*T. Alternatively, a separate, heterologous gene regulator in *P. fluorescens* could be acting to induce *pdu* gene expression in the absence of 1,2 PD (or acting in combination with altered PocR and Crp activity) in this species. Such observations regarding altered expression and activity across different species are an intriguing source of further study with R995 + *pdu S*T.

We observed robust expression and isolation of Pdu MCPs from R995 + *pdu S*T strains in a range growth media, and we found that MCP isolation can be achieved in a mini‐prep format using a tabletop microfuge. In addition, we found that the presence of 1,2 PD and protease inhibitor during the purification protocol is not necessary to achieve successful MCP isolation. These results indicate the potential for convenient flexibility and utility of the R995 + *pdu S*T clone (or similar MCP gene clones) for future applications across different bacteria. Such applications could possibly include providing useful metabolic options for desired species or enabling MCP‐based nanobiotechnology across bacteria. Strategies and approaches towards these ends will be developed for future studies involving this clone.

## Experimental procedures

### Bacterial strains and plasmids

Please refer to Table 1 for a list of strains and plasmids used in this study. For routine growth of strains for cloning and maintenance, either Lennox broth (LB) or M9 minimal media were used (Lennox, 1955; Green and Sambrook, 2012). Antibiotics were used at the following concentrations (μg ml^−1^): kanamycin 50, tetracycline 5, rifampicin 75, nalidixic acid 5, ampicillin 200, chloramphenicol 10.

**Table 1:** Strains and plasmids used in this study

Strain	Species	Comments
χ3477	*Salmonella* Typhimurium	Gift from Roy Curtiss III
χ3477 Δ*pdu*	*Salmonella* Typhimurium	This study
TOP10 Rif	*Escherichia coli*	Rif‐R isolate of Invitrogen strain
TOP10 Nal	*Escherichia coli*	Nal‐R isolate of Invitrogen strain
ATCC 43975	*Salmonella bongori*	American type culture collection
ATCC 13883	*Klebsiella pneumoniae*	American type culture collection
ATCC 29544	*Cronobacter sakazakii*	American type culture collection
ATCC 14041	*Serratia marcescens*	American type culture collection
ATCC 13525	*Pseudomonas fluorescens*	American type culture collection
PAK *pilA*	*Pseudomonas aeruginosa*	Wilson *et al*. (2007)
ATCC 51812	*Pseudomonas sp. 51812*	American type culture collection
ATCC 49128	*Pseudomonas putida*	American type culture collection

Plasmid	Comments
R995	Pansegrau *et al*. (1994)
R995 Tc	Santiago *et al*. (2011)
R995 + *pdu S*T	This study
pKD3	Datsenko and Wanner (2000)
pKD4	Datsenko and Wanner (2000)
pKD46	Datsenko and Wanner (2000)
pCP20	Datsenko and Wanner (2000)

### Cloning of the *S*. Typhimurium *pdu* and *cob/cbi* genes using FRT‐Capture

The region targeted for cloning via FRT‐Capture is diagrammed in Fig. 1A (Santiago *et al*., 2011; Wilson *et al*., 2013). Using primers listed in Table S1, we designed PCR products containing single FRT sites to insert at regions flanking the S. Typhimurium *pdu*/*cob*/*cbi* region (in the *erfK* and *yeeA* genes) (Fig. 1A,B). This was achieved using standard recombineering reagents and techniques (Datsenko and Wanner, 2000). Plasmid R995 Tc (containing a single FRT site) was introduced into this strain followed by introduction of plasmid pCP20 which expresses the FLP recombinase (Datsenko and Wanner, 2000; Santiago *et al*., 2011). This resulted in excision of the *pdu/cob/cbi* gene segment and insertion of this segment into R995 Tc, and this construct could be isolated using conjugation to a fresh *E. coli* background containing a counterselection (such as TOP10 Rif‐R or TOP10 Nal‐R) (Fig. 1B). PCR was used to confirm successful cloning and conjugation of the targeted genomic segment (Fig. 1C). The strain *S*. Typhimurium Δ*pdu* was obtained as a by‐product of this procedure as this strain was easily isolated after FLP treatment (by screening pCP20‐cured isolates for Km‐S and Cm‐R).

### Transfer of R995 + *pdu S*T to Gram‐negative recipients

An *E. coli* strain containing R995 + *pdu* ST was used as a donor in conjugative transfer to various Gram‐negative recipients as described previously (Wilson *et al*., 2004; Wilson and Nickerson, 2006). All transconjugants were selected on M9 medium (since the donor *E. coli* strain is auxotrophic) containing Tc and Km, except the conjugation to *S*. Typhimurium Δ*pdu* which was selected on LB Cm Tc Km. Plasmid DNA was isolated from each strain to verify transfer via PCR (Fig. 1C).

### Testing of R995 + *pdu S*T gene expression

MacConkey media containing 1,2 PD as a carbon source were formulated as follows (per litre): 20 g Bacto peptone, 5 g NaCl, 10 ml 1,2 PD (Sigma #398039), 75 mg neutral red, 15 g Bacto agar. Coenzyme B_12_ (Sigma #CO884) was added as indicated to a concentration of 500 nM. Stock solutions and agar plates containing coenzyme B_12_ were stored protected from light. RT‐qPCR was performed as described previously (Wilson *et al*., 2008; Soni *et al*., 2014; Herman *et al*., 2016) using total RNA harvested from the indicated strains (grown either with or without 1,2 PD) which was then converted to cDNA and analysed using *pduW* and *korB* primers indicated in Table S1.

### Isolation and analysis of MCPs expressed from R995 + *pdu S*T

We modified a commonly referenced Pdu MCP isolation protocol (Sinha *et al*., 2012) as follows: strains containing R995 + *pdu S*T or R995 vector were routinely grown overnight in 100 ml of Pdu medium (1× M9 salts, 1 mM MgSO4, 0.5% succinate, 0.5% 1,2 PD, 2 mg ml^−1^ casamino acids) containing Km and Tc selection. Seventy millilitres (35 ml in each of two Oak Ridge tubes) of the cultures was centrifuged at 12 000 *g* to pellet the cells, the supernatant was entirely removed, and the pellets in each tube were resuspended in 7.5 ml buffer A (50 mM Tris‐HCl pH = 7.5, 500 mM KCl, 12.5 mM MgCl2, 0.5% 1,2 PD, 5 mM beta‐mercaptoethanol, 0.4 mM protease inhibitor AEBSF [Sigma #A8456], 100 μg ml^−1^ lysozyme [Sigma #L3790], 2 units ml^−1^ DNase I [New England Biolabs #M0303S]) in each tube. The starting amount of cells for each prep was typically between 6.3 × 10^10^ and 1.4 × 10^11^ CFU total, determined by serial dilution and plating of samples for CFU counts (or using OD600 measurement correlated to known CFU amounts for each strain). Three millilitres of B‐PER lysis reagent (Thermo Fisher #90084) was added to each tube and mixed gently, and the tubes were incubated with rotation at room temperature for 1 h. The samples were centrifuged at 12 000 *g* for 5 min to pellet cell debris, the supernatant transferred to fresh tubes, and the samples then centrifuged at 20 000 *g* for 30 min to pellet MCPs. After careful removal of the entire supernatant, the pellets were resuspended and combined in a total volume of 500 μl buffer B (50 mM Tris‐HCl pH = 7.5, 50 mM KCl, 5 mM MgCl2, 1% 1,2 PD, 0.4 mM AEBSF) and transferred to a single microfuge tube. The sample was then centrifuged at highest speed in a microfuge (16 000 *g*) for one minute to pellet any additional insoluble material, and the supernatant was transferred to a fresh microfuge tube (which represented the final sample). For analysis, we would typically load 1%–3% of total yield on a 12% SDS‐PAGE gel followed by Coomassie Blue staining, with total yield being typically between 500 and 2000 micrograms of protein. To identify proteins from SDS‐PAGE gels, bands were excised from the gels and subjected to Nano‐LC‐MS/MS mass spectrometry analysis (Alphalyse, Inc., Palo Alto, CA). Briefly, the proteins obtained from excised gel bands were reduced, carbamidomethylated, and subsequently digested with trypsin. The resulting peptides were concentrated via lyophilization and redissolved for injection into a Dionex nano‐LC system followed by MS/MS analysis on a Bruker Maxis Impact QTOF instrument. The MS/MS spectra were used for Mascot‐based searching of UniProt and NCBI protein databases containing more than 80 million known non‐redundant protein sequences. For the MCP mini‐preps, strains were grown overnight with plasmid selection in 25 ml of media, and then 10 ml of culture was evenly distributed across eight microfuge tubes and pelleted in a microfuge at 16 000 *g* for 5 min at 4°C. After complete removal of the supernatant via aspiration, 500 μl of buffer A containing B‐PER was added to each tube, and the cells were fully resuspended. After rotation at room temperature for 45 min, the samples were microfuged at 12 000 *g* for 3 min at 4°C to pellet cell debris. After this spin, the supernatants for each sample were pooled into four microfuge tubes (now containing approximately 1 ml each), and the samples were microfuged at 16 000 *g* for 1 h at 4°C. After this spin, the supernatants were removed, and the pellets were resuspended and pooled in a total of 150 μl of buffer B. Typical total protein yield from the mini‐prep was 100–200 μg. All MCP preps were stored at either 4°C or at −80°C.

### Electron microscopy

Transmission electron microscopy (TEM) was performed using standard protocols as described previously (Jennings *et al*., 2012). MCP samples were spotted onto carbon‐coated copper grids (300 mesh), negatively stained using 4% uranyl acetate and visualized using an Hitachi H‐7600 TEM microscope at 80 kV. To quantify MCP size, the diameters of at least 50 individual MCPs were measured in random fields of view in TEM images for the indicated species. The widest aspect of a given MCP diameter was measured for this analysis.

### Different media used for MCP growth and isolation

The different growth media tested for support of MCP expression and isolation from R995 + *pdu S*T strains are as follows (ingredients given per litre): Pdu medium (recipe provided above), Lennox LB broth (10 g tryptone, 5 g yeast extract, 5 g NaCl), tryptic soy broth (TSB) (15 g tryptone, 5 g soytone, 5 g NaCl), 2xYT broth (16 g tryptone, 10 g yeast extract, 5 g NaCl), PYE broth (10 g peptone, 10 g yeast extract, 5 g NaCl), CAYE broth (20 g casamino acids, 10 g yeast extract, 5 g NaCl), nutrient broth (NB) (4.5 g beef extract, 7.5 g peptone, 5 g NaCl), Mueller Hinton broth (MH) (17.5 g casein acid hydrolysate, 3 g beef extract, 1.5 g starch). All media were supplemented with 1,2 PD to 0.5%. Control strains containing the R995 vector grown in these media did not display the MCP bands via SDS‐PAGE and Coomassie staining (data not shown).

## Conflict of interest

None declared.

## Supporting information


**Table S1.** DNA primers used in this study.
**Table S2.** MCP yields from R995 + *pdu S*T‐containing bacteria.
**Fig. S1.** The utilization of 1,2 PD directed by R995 + *pdu S*T depends on coenzyme B12.
**Fig. S2.** Diameters of MCPs isolated from different bacterial species containing R995 + *pdu S*T.
