# Supplementary material for: Transfer and analysis of Salmonella pdu genes in a range of Gram‐negative bacteria demonstrate exogenous microcompartment expression across a variety of species
Source: Microb Biotechnol. 2017 Oct 2;11(1):199–210. doi: 10.1111/1751-7915.12863 (PMC5743805; doi:10.1111/1751-7915.12863)
Supplement: Supplementary file 1 — Table S1. DNA primers used in this study. Table S2. MCP yields from R995 + pdu ST‐containing bacteria. Fig. S1. The utilization of 1,2 PD directed by R995 + pdu ST depends on coenzyme B12. Fig. S2. Diameters of MCPs isolated from different bacterial species containing R995 + pdu ST. [file MBT2-11-199-s001.pdf]

<u>Supplemental Table S1</u>: DNA primers used in this study.

<u>*erfK* insertion (PCR template = pKD4)</u>

agcctgtcacaatcgccgtaccgcagaacaatactcagccctactgggctatctggacaaggg

aggccaggcctgcccgcgctaatgttgaccggcattccggcatatgaatatcctccttagttcc

<u>*yeeA* insertion (PCR template = pKD3)</u>

ccgataagtcattaagcccttttgaaatacggttgtaccgatttaaatggcgcgccttacgcc

tgctgctcgttcattagctgccgcaactcggcagcaatatcatatgaatatcctccttagttcc

<u>*pduW*</u>

tatggcagatgcgcaggtgacaattaagac

tgacaacaaatcacccgtaatgcgctgagt

<u>*cobU*</u>

acctcatccgccgctgccgccagtcgttgg

cttaattggcgatgcgccgcaggtactgta

<u>*traC*</u>

tcgtggccgatgcccagctcgtcgcccagg

cccgcgctggatgacctacaagcaggccgc

<u>*korB*</u>

gacctggacctgatcgacgaagatccgc

tgatttcctgggtgtcgtcgtcaagccac

Supplemental Table S2: MCP yields from R995 + *pdu S*T-containing bacteria

Background strain (containing R995 + *pdu S*T)	Total MCP yield (micrograms)*
S. Typhimurium Δ*pdu*	1521.86
S. bongori	1517.89
E. coli	1470.17
C. sakazakii	1489.06
K. pneumoniae	1533.79
P. fluorescens	1463.22

*representative data from preps obtained from 70 ml of LB cultures supplemented with 1,2 PD as described in Materials and Methods

Supplemental Figure S1

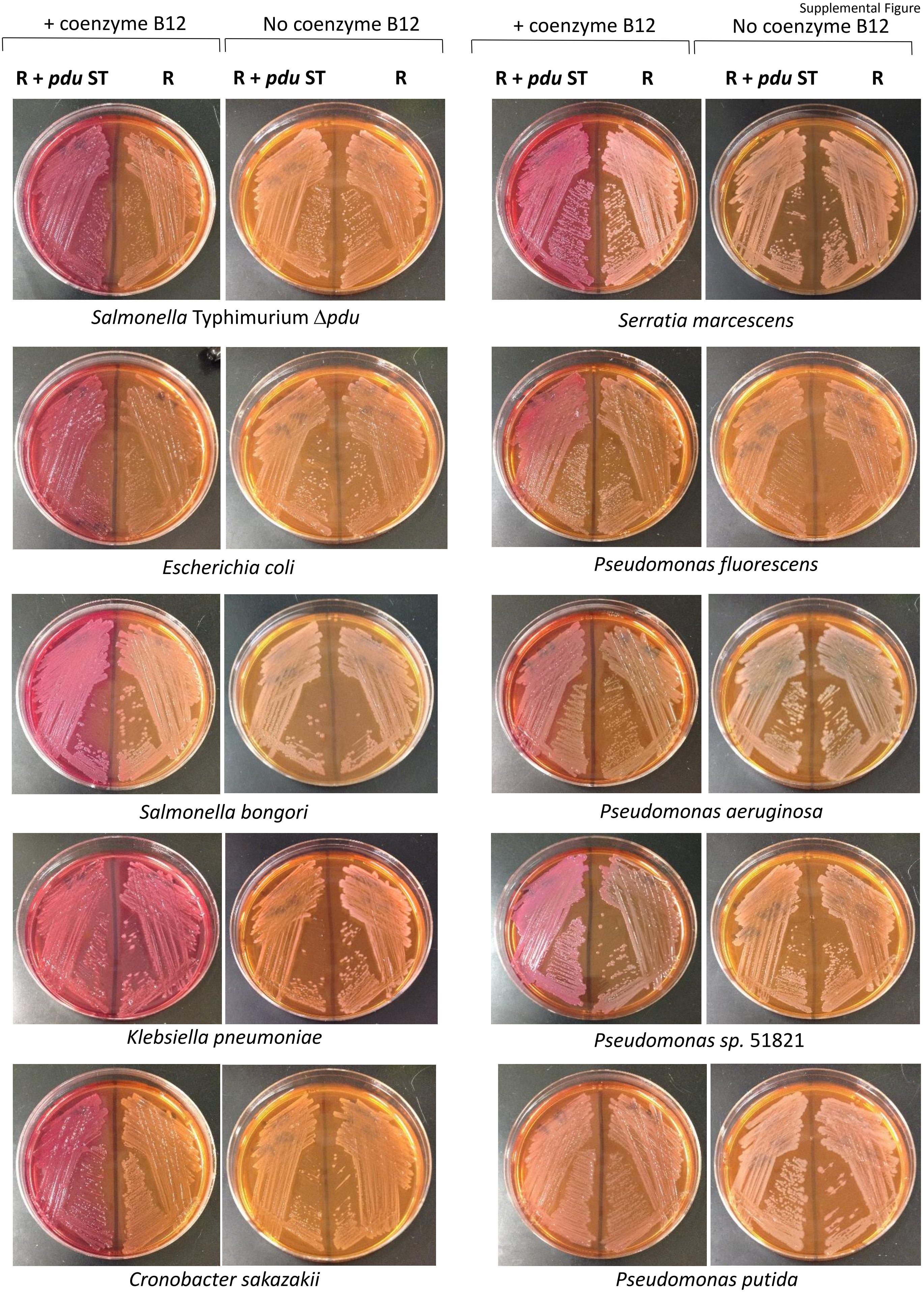

Supplemental Figure S1. **The utilization of 1,2 PD directed by R995 + *pdu S*T depends on coenzyme B12**. The indicated bacterial species containing either R995 + *pdu S*T or the R995 vector control were streaked onto MacConkey medium supplemented with 1,2 PD and coenzyme B12 or with 1,2 PD in the absence of coenzyme B12. Utilization of 1,2 PD will result in a pink/red colony color on this medium.

Supplemental Figure S2

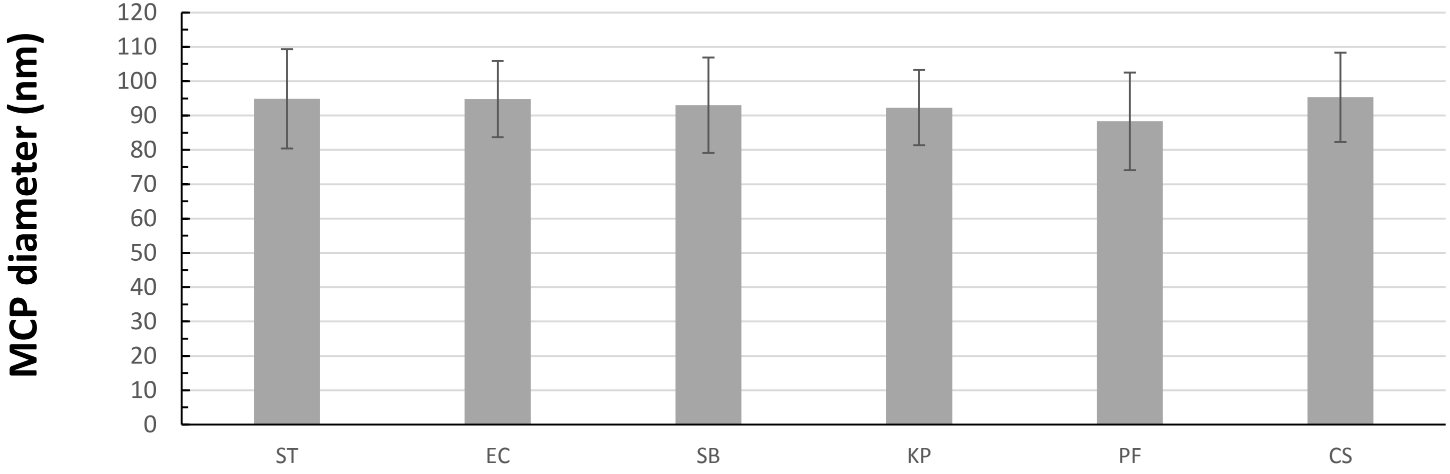

Supplemental Figure S2: Diameters of MCPs isolated from different bacterial species containing R995 + *pdu S*T. The diameters of at least 50 individual MCPs from the indicated bacteria containing R995 + *pdu S*T were measured in random fields of view in TEM images. The widest aspect of a given MCP diameter was measured for this analysis. The average and standard deviation for each sample is graphed. Abbreviations: ST = *S.* Typhimurium Δ*pdu*; EC = *E. coli*; SB = *S. bongori*; KP = *K. pneumoniae*; PF = *P. fluorescens*; CS = *C. sakazakii*.
